# Prestige Fetishism in the Academy: Comte's Mirror, the Magic Mirror or an Illusion of Reality?

**DOI:** 10.1111/1468-4446.13224

**Published:** 2025-05-05

**Authors:** Jian Wu

**Affiliations:** ^1^ School of Social Sciences Lecturer in Education Keele University Newcastle‐under‐Lyme UK

**Keywords:** academic class fragmentation, commodity fetishism, prestige fetishism, scholarship as struggle, unviersity rankings

## 1 Introduction

The call for commentary frames *Scholarship as Struggle: Stories of Censorship, Marketisation, and Resistance* as ‘the ongoing managerial and ideological attacks on higher education’ (BJS (British Journal of Sociology) 2024). This assertion is undoubtedly accurate whether viewed through the lens of the rise of neoliberalism since the 1980s with its accompanying discourses of new public management or the rule of the market (Olssen and Peters 2005) or of the more recent right‐wing populist assault against ‘“liberal elites” [from higher education] in the name of tradition or nation’ (Dillabough 2022, 183). However, this framing is also problematic as it depicts academics as passive victims—erasing their role in participating in, reinforcing, or benefiting from the system, disregarding the internal academic power structures that shape higher education (HE), and externalising resistance as a task that should be directed towards the system rather than serving as a reflection on their professional practice. Take the example of audit culture and the rise of ‘impact’, which, in the UK, is often attributed to the Research Excellence Framework 2014 (Pearce and Evans 2018). According to Apple (2005), these phenomena are not ‘totally reducible to the needs of neo‐liberals and neo‐conservatives’ (20), and he encourages a more nuanced understanding of class relations and class projects to fully grasp them. However, his class analysis remains confined to the friction between the academic and new managerial classes, overlooking the academic community as a site of class struggle among academics themselves.

Bourdieu (2004, 4) uses the ‘mirror effect’ metaphor to illustrate how reflexivity involves not mere self‐awareness but a critical recognition of how one's position within a social hierarchy influences perceptions, behaviours, and academic or professional engagement. Thus, Bourdieu's ‘mirror’ encourages us to closely examine our academic identity and reflect on our own practices. In this spirit, I maintain that, despite the straitjacket of external factors like marketisation imposed on the academy, academics must still scrutinise the extent to which they have—consciously and unconsciously—contributed to the maintenance of ‘the rhythm of the [capitalist] iron system’ (Horkheimer and Adorno 1997, 120).

Based primarily on my experiences, observations, and reflections as a PhD student and later as an academic in the UK, I argue that although grouping people into a set of hierarchical social categories may be inevitable in any organisation, social relations between universities and society at large and among academics appear increasingly defined by the sheer properties and purported values of prestige indicators in the forms of perceived quality, status, and reputation ‐ often measured through rankings, evaluations, and bibliometric indicators (Musselin 2018). This prestige fetishism not only leads to an illusion of reality about HE and its intrinsic values but also causes class stratification and destroys solidarity within the academy. In the following pages, I will begin by defining prestige fetishism and describing its corrosive pervasiveness. I will then explain how it generates a false perception of reality regarding education and research. Finally, I will elaborate on how it causes class stratification and fragments solidarity within the academy, hindering the formation of a unified class consciousness for change.

## 2 Prestige Fetishism

Marx defines commodity fetishism as a ‘phantasmagoric form of a relation between things’ (Marx 1976, 165), describing how the inherent value of commodities is attributed solely to commodities per se, obscuring (and consequently, denigrating) the social relationships and labour that produce them. Theorists from the Frankfurt School broadened commodity fetishism beyond commodity production with the concept of reification, arguing that capitalism extends this ‘thingification’ to all aspects of life, including politics, law, education, and even consciousness (Rose 2024). They contend that capitalism remains resilient in the face of revolutionary changes despite the crises predicted by Marxist theory because of reification, which transforms social relations into relations between things, legitimising the system by concealing capitalism's structural contradictions, such as the incommensurability of class interests between capitalists and the proletariat. In other words, ‘the illusions that arise out of commodity fetishism … are necessary and real [for the functioning of a capitalist system], but nevertheless, they are illusions’ (Rose 2024, 17).

For this commentary, prestige fetishism denotes the reification or thingification of prestige—originally a social construct that is relational and context‐dependent—into a static and abstract notion that lacks educational, social, and intellectual contexts and considerations. In other words, this thingification influences all facets of HE, including academic labour, academic class consciousness, and the relationship between the academy and wider society because the notion of prestige in the academy gradually detaches from the social value and operational form of HE, typified by intellectual engagement and truth‐seeking activities—fraught with idiosyncrasies and subjectivities—which are hard to measure and impossible to rank. Instead, academics perceive their colleagues not as co‐producers of knowledge but rather as prestige indicators, such as through their citation counts. For example, the pervasive rhetoric of ‘excellence’ across the academy spurs hyper‐competition that is antithetical to collaborative endeavours (Moore et al. 2017). Similarly, students increasingly frame university choice around employability and reputational factors linked to rankings, rather than academic or civic missions, as they take on the role of consumers of higher education (Gupta et al. 2025).

## 3 The Pervasiveness of Prestige Fetishism

Within this zeitgeist of prestige fetishism, education loses its social dimensions and becomes just a ‘thing’ that is captured crudely by *prestige indicators* such as awards, accolades, rankings, or professional member affiliations. When these indicators are eviscerated of intrinsic values such as intellectual contributions and detached from the socio‐relational contexts that make them possible, they simply become trophy indicators that are performative and status‐driven and do not necessarily reflect genuine quality or value. I will illustrate this proposition with three examples: university rankings, the UK's immigration policy, and academic labour.

**FIGURE 1:**
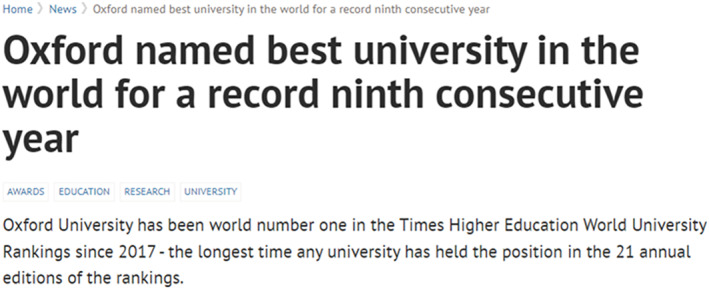
Oxford ranking news on its website. https://www.ox.ac.uk/news/2024‐10‐09‐oxford‐named‐best‐university‐world‐record‐ninth‐consecutive‐year.

The superlatives ‘best’ and ‘longest’—along with the unparalleled qualities of ‘world number one’ and ‘record ninth consecutive year’—used in the heading and lead paragraph in the celebratory news of Oxford's ranking by the Times Higher Education World University Rankings (Times Rankings) are a display of prestige fetishism (Figure 1) that sought to highlight the ‘exceptional’ quality of Oxford as an academic institution. We could easily and quite justifiably attribute this hubris to the ‘invisible hand’ of the market, conceptualising the ranking epidemic as a mimetic ritual in which universities have no choice but to participate. However, it raises the question of the unthinkingness about rankings by the world's supposedly ‘number one’ university, for whom the superlatives—meaningless though they may be—matter more than more thoughtful questions such as how possible it is to rank universities. A separate and low‐tiered prestige ecosystem exists for less elite universities, be it ‘best university voted by students’, ‘employers’ choice’, or ‘excellence in teaching quality’. If these titles do not suit them, other categories such as ‘sustainability’, ‘young universities’ or ‘regional universities’ are up for grabs. Prestige of various kinds is stripped of its complexity and relational dimensions but pursued for its own sake.

It is not only universities that fetishise prestige. The UK's High Potential Individual visa scheme aims to attract recent graduates from globally ‘top‐ranked’ universities. It allows recipients to stay in the UK for up to 2 years (three for PhDs) to seek employment or pursue entrepreneurship without sponsorship. Being *top‐ranked* means being concurrently in the top 50 of any two of the ranking systems from Times Rankings, Quacquarelli Symonds World University Rankings, and The Academic Ranking of World Universities. In 2022, the Shanghai Municipality in China introduced a similar talent programme, including universities from the U.S. News Rankings. Individuals are not valued for their intrinsic abilities, potential, or diverse experiences but are selected for an association with a brand value. Such distorted schemes not only alienate other ‘high potential’—variously defined—candidates but, more importantly, denigrate the social worth and dignity of humans. Moreover, such schemes illustrate how national policies, such as immigration regimes, are closely intertwined with the prestige economy of global HE.

Academic labour is increasingly reified in esteem indicators such as the reputation of research funding agencies, impact factor of journals, number of citations, and even invitations for keynote speeches. In a way, we have all become what Pardo‐Guerra (2022) calls 'quantified scholars,’ gradually detaching from intellectual contribution and reattaching to metric proxies. Essentially, ‘esteem’ makes prestige sound less pretentious without creating much ontological difference. Intellectual contributions of academics are transformed into externalised indicators of value, ultimately simplified into prestige, a symptom of fetishism. Of course, not all metrics are bad, but they become dangerous when their meanings rely solely on the perception of prestige hierarchy. A recent study on the publication preferences of health and medical researchers revealed that a journal's impact factor is a predominant influence on these researchers' behaviours: their strongest preference was for the highest impact factor, followed by a moderate impact factor; some respondents were even prepared to omit results in exchange for a higher impact factor (Bohorquez et al. 2024).

## 4 Reality Inversion

**FIGURE 2:**
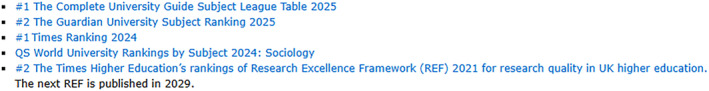
University of cambridge sociology department. https://www.sociology.cam.ac.uk/about.

The single‐minded fixation on prestige not only damages research quality, as illustrated by the previous example, but also constructs a false reality, akin to how commodity fetishism represents an economic manifestation of the broader philosophical issue of appearance versus reality (Burke 1979). University College London's Institute of Education (IOE) has displayed a banner outside its building for many years, claiming it is the world's Number 1 for Education. The claim inherent in this assertion is that if someone studies education at IOE, they will receive the ‘best’ education. Are the academics who do social research, especially in education within IOE, unaware of the inherently unrankable nature of educational intellectual pursuits? Of course, they are aware. Therefore, it is not vituperative to assume that IOE is misleading (if not entirely deceiving) both itself and the public, including students and potential applicants, by predicating institutional credibility on this untenable claim and utilising it as a commercial assertion to its advantage. In essence, being number 1 is perceived to capture all the qualities of an education. Casting an eye on the Cambridge Sociology website (Figure 2), I wonder what those sociologists make of their department's ‘prestige’. Or are they coerced by the university to show off their standings? If not, where have all their theoretical vigour, empirical testing, proclivity for critique, and decolonising activism gone? My unkind guess is that, even for them, the best public‐facing ‘thing’ is the department's prestige; whether they truly believe in it or not does not matter that much. It is not hard to see that ‘fetishism is not only an inverted representation of reality, but also an *inversion of reality itself’* (Jappe quoted in Mau [2023, 393], original emphasis). Nevertheless, social actors organise their actions in conformity with this illusion of reality.

## 5 Class Stratification and Solidarity Fragmentation

The reification of something ‘involves missing its human and social characteristics and its amenability to social control, together with an apprehension of its merely objective, indifferent, independent, abstract, possibly alien or extraneous features’ (Burke 1979, 79). Consequently, the reification of prestige fetishism transforms subjects (people) into objects (academic commodities) and objects into subjects, thereby making the objects the factor determining the nature of the social relationship between people. This lack of ‘human and social characteristics’ and capitulation to ‘extraneous features’ of academic work manifested in reactions to the Times Rankings and Stanford's list of the world's top 2% most cited scientists released in 2024 when I encountered this call for commentary. Many universities instantly took to social media to publicly laud their achievements, whether those were their overall ranking or some narrower accolade such as Asia Rankings or individual subject rankings. In a similar manner, congratulatory posts about the world's top 2% quickly appeared across institutional and personal websites and social media platforms, artfully sprinkled with humble‐brag tweets from the scientists themselves, thanking those who helped them reach this milestone. In contrast to Bourdieu's ‘mirrors’, the more fitting mirror metaphor for these types of reflexivity might be Comte's mirror (located in the room where he coined the term ‘sociology’), into which he could gaze to admire himself after completing a sentence (Lepenies 1998; Back 2016), or the Magic Mirror in Snow White, which the Evil Queen consults for validation of her beauty.

These two metaphoric mirrors tellingly reveal the stratified nature of academic classes (the elite vs. the rest) and highlight the reified and exclusionary nature of ‘prestige’, gaslighting academics and the public into believing that prestige is the sole and definitive marker of academic pursuit and worth. During this process, knowledge is pursued for instrumental reasons rather than for its own sake, while the Humboldtian ideal of the university as a community of scholars and students is cast aside. Two consequences follow from this reification process.

First, it exacerbates class distinctions among academics, transforming their relationship to class competition. While early‐career researchers want to climb the prestige ladder to stay in the academy or gain promotions, senior academics keep accumulating ‘esteems’ for fear of losing their advantageous position. Consequently, exploitation becomes an inherent feature of a stratified academy, as typified by hiring fixed‐term teaching staff to relieve permanent staff from teaching obligations so they find time to do research, a key measure of academic prestige. Since prestige thrives on a structure and strategy of hierarchy and exclusion, it, in turn, produces a spectrum of academic classes—for example, endowed chair professors, rising stars, tenured (permanent) academics, fixed‐term teaching fellows, and adjunct lecturers—whose survival and growth depend largely on the prestige goods they can accumulate. Thus, there is no ‘we’ as a unified collective in academia since different academic classes compete with each other under the compulsion of prestige drive. The class fault lines also occur at a transnational level. A new study reveals that academics in the Global South are twice as likely to be promoted to professor based on their publication volumes as are those at HE institutions in the Global North (Lim et al. 2025). This obsession with metrics in the Global South underscores the existing regional disparity between the transactional professional classes and suggests a potential exacerbation of the class divide in that region.

The Frankfurt School's emphasis on the subjective aspects of reification—specifically, how individuals experience it, how it hinders their understanding of society, and the social forces shaping their lives (Rose 2024)—speaks directly to the second consequence. The naturalisation and internalisation of prestige fetishism lead people to become incognizant of the systemic oppressive social structures and, concomitantly, the dissolution of their subjectivity and social agency. The reality‐distorting and consciousness‐inhibiting effects of prestige fetishism, compounded by the absence of meaning and relatable social relationships, produce conformism, the pursuit of self‐interest, and passivity among members of the academic community and distract them from questioning the exploitative mechanisms of neoliberalism, some of which are self‐reinforced. Furthermore, the culture fostered by the obsession with prestige alienates different academic classes from the intrinsic values of academic work and, more importantly, their own power of agency by rationalising and mystifying domination, exploitation, and self‐exploitation. Consequently, they overlook the conditions that give rise to an oppressive system that thwarts rather than serves their interests as well as the prerequisite for systemic change and genuine emancipation—a unified class consciousness.

## 6 Conclusion

HE is a place full of ironies. It preaches about the values of intellectual engagement while reducing that engagement to a commodity of exchange. It preaches about diversity, inclusion, and equality while never stopping trying to create a hierarchical, exclusionary system. Prestige indicators crudely reduce the diverse types and qualities of institutions, academics, and knowledge to a simple ordinal ranking, reifying them into mere ‘things’ that are disconnected from social relationships or any context. Institutions and academics need to rise to the occasion to transcend this simplistic and immature fetishism for prestige, which has been solidified as an object of their conscious and unconscious. This is easier said than done, especially for lesser‐known institutions or unestablished academics, because they need to look to their more ‘successful’ peers who are, in a way, their ‘mirrors’—in a manner similar to Luhmann's second‐order observation whereby they imitate how the ‘successful’ perceive, interpret, and communicate about the phenomenon of prestige fetishism.

The ongoing crisis in the UK HE sector, marked by severe financial strain and widespread staff layoffs, is intensifying academic class fragmentation at inter‐ and intra‐institutional as well as inter‐discipline levels. The divide between research‐intensive (i.e., prestige‐ and income‐generating) academics, early‐career researchers, and fixed‐term staff, along with the divide between financially viable and unviable disciplines, is further stratifying the class structure. I do not take neoliberalism's structural forces for granted, nor do I believe we are entirely deprived of agency. Preventing universities from becoming ‘brutally philistine’ and ‘self‐avowed service stations for the capitalist economy’ (Eagleton 2024, 3) depends on empowering all those working in the academy to think of themselves as citizens in a shared public life. To combat prestige fetishism as an effort to undermine the neoliberal capture of our life and identity, so‐called world‐class universities and most‐cited researchers, without whose proactivity neoliberal performativity would not have flourished, should shoulder a more significant share of responsibility, given the weight and impact they have on the sector. Theoretically, they should look into Bourdieu's mirror to reflect on their identity and duties to the community. At the practical level, they should stop engaging in activities that promote prestige fetishism. In placing the blame for the current HE crisis solely on capitalism, neoliberalism, and university administrators, we, academics, absolve ourselves of complicity.

## Conflicts of Interest

The author declares no conflicts of interest.

## Data Availability

Data sharing is not applicable to this article as no new data were created or analysed in this study.
